# Stanene-hexagonal boron nitride heterobilayer: Structure and characterization of electronic property

**DOI:** 10.1038/s41598-017-16650-5

**Published:** 2017-11-27

**Authors:** Asir Intisar Khan, Trisha Chakraborty, Niloy Acharjee, Samia Subrina

**Affiliations:** Department of Electrical and Electronic Engineering, Bangladesh University of Engineering and Technology (BUET), Dhaka, 1205 Bangladesh

## Abstract

The structural and electronic properties of stanene/hexagonal boron nitride (Sn/h-BN) heterobilayer with different stacking patterns are studied using first principle calculations within the framework of density functional theory. The electronic band structure of different stacking patterns shows a direct band gap of ~30 meV at Dirac point and at the Fermi energy level with a Fermi velocity of ~0.53 × 10^6^ ms^−1^. Linear Dirac dispersion relation is nearly preserved and the calculated small effective mass in the order of 0.05m_o_ suggests high carrier mobility. Density of states and space charge distribution of the considered heterobilayer structure near the conduction and the valence bands show unsaturated π orbitals of stanene. This indicates that electronic carriers are expected to transport only through the stanene layer, thereby leaving the h-BN layer to be a good choice as a substrate for the heterostructure. We have also explored the modulation of the obtained band gap by changing the interlayer spacing between h-BN and Sn layer and by applying tensile biaxial strain to the heterostructure. A small increase in the band gap is observed with the increasing percentage of strain. Our results suggest that, Sn/h-BN heterostructure can be a potential candidate for Sn-based nanoelectronics and spintronic applications.

## Introduction

Of late, group-IV two dimensional (2D) materials such as silicene, germanene and stanene have received special attention attributed to their similar electronic properties with the graphene and intriguing prospects in the nanoelectronic device applications^1^. In fact, stanene, a buckled honeycomb structure of 2D hexagonal tin film, has drawn keen interest due to its intriguing prospect as quantum Hall insulator^2^, topological insulator^3^ and topological superconductor^4^. In addition, stable low buckled form of stanene^5–7^, synthesized on the Bi_2_Te_3_ (111) substrate^8^ is theoretically investigated to support a large-gap 2D quantum spin Hall (QSH) state at room temperature and enables superior electric conduction with zero dissipation^9,10^. However, stanene has a zero band gap without spin orbiting coupling (SOC) while ~0.1 eV SOC induced band gap has been reported in the literature^11^. This zero band gap feature limits the use of stanene in the high performance semiconductor based nanoelectronic devices such as field effect transistor^12^. In this context, Garg *et al*.^13^ has recently reported the opening of a band gap in stanene by the patterned boron-nitride (BN) doping in stanene using density functional theory (DFT). In addition, Chen *et al*.^14^ has studied graphene-stanene heterobilayer models with several stacking methods and reported a band gap of ~77 meV for the most stable configuration. Furthermore, graphene/hexagonal boron nitride (graphene/h-BN) bilayer has been reported to show a small band gap at Dirac point in contrast to the zero band gap of graphene monolayer as well as higher carrier mobility than the graphene bilayers^15–17^. In fact, chemical stability and significant band gap of the monolayer h-BN^16,18^ make it promising for heterostructure based nano-devices.

Hence, following the deep scientific interest in modulating the band gap and other electronic properties while preserving the Dirac cone of stanene^5^, we report the structural and electronic properties of stanene/hexagonal boron nitride (Sn/h-BN) heterobilayer using first-principles density functional theory (DFT) calculations. A band gap opening along with the appearance of nearly linear relation near the Dirac point is observed in the proposed heterobilayer structure. Three representative models of the hybrid structure based on different stacking are investigated in terms of their stability, electronic property such as band structure, density of states, real space charge density and effective mass as well as the modulation of band gap under tensile strain and varying the interlayer distance. Our proposed structures with a finite band gap and high carrier mobility would provide further insight and encouragement on the modeling of stanene-based nanoelectronic and spintronic devices.

## Methods

The electronic properties of the proposed heterobilayer structure are investigated using the density functional theory (DFT) with a plane-wave basis set using the Ab initio code PWSCF package of Quantum Espresso^19^. The electron-ion interactions are accounted using norm conserving Troullier Martin pseudo-potentials^20^. To describe the electron exchange correlation energy, the generalized gradient approximation (GGA)^21^ with Perdew-Burke-Ernzerhof (PBE) exchange correlation functional is implemented. In order to consider the Van der Waals (vdW) inter-molecular attractive forces, we have used DFT-D2^22^, an empirical dispersion correction proposed by Grimme^23^ throughout the simulations. For the structural optimization, the plane wave basis cut off is set at 550 eV (40 Ry) with a convergence threshold on force of 10^−4^ Ry/a.u. The first Brillouine zone of the unit cell is sampled with a 12 × 12 × 1 Monkhorst-Pack grid for the geometry optimization and a 15 × 15 × 1 grid for the subsequent calculations. The interaction between the two adjacent bilayers is eliminated by introducing a sufficient vacuum of 20 Å along the direction perpendicular to the surface.

The effective masses of electrons $({m}_{e}^{\ast })$ and holes $({m}_{h}^{\ast })$ have been calculated from the curvature of the conduction band minimum and the valence band maximum respectively at the Dirac point for the three (3) configurations of stanene/h-BN heterobilayer using the following formula:

1 $${m}^{\ast }={\hslash }^{2}\,{[\frac{{\partial }^{2}E(k)}{\partial {k}^{2}}]}^{-1}$$

where, m* is the particle effective mass, E(k) is the dispersion relation, k is the wave vector and ћ is the reduced Planck constant.

## Results and Discussions

**Figure 1:**
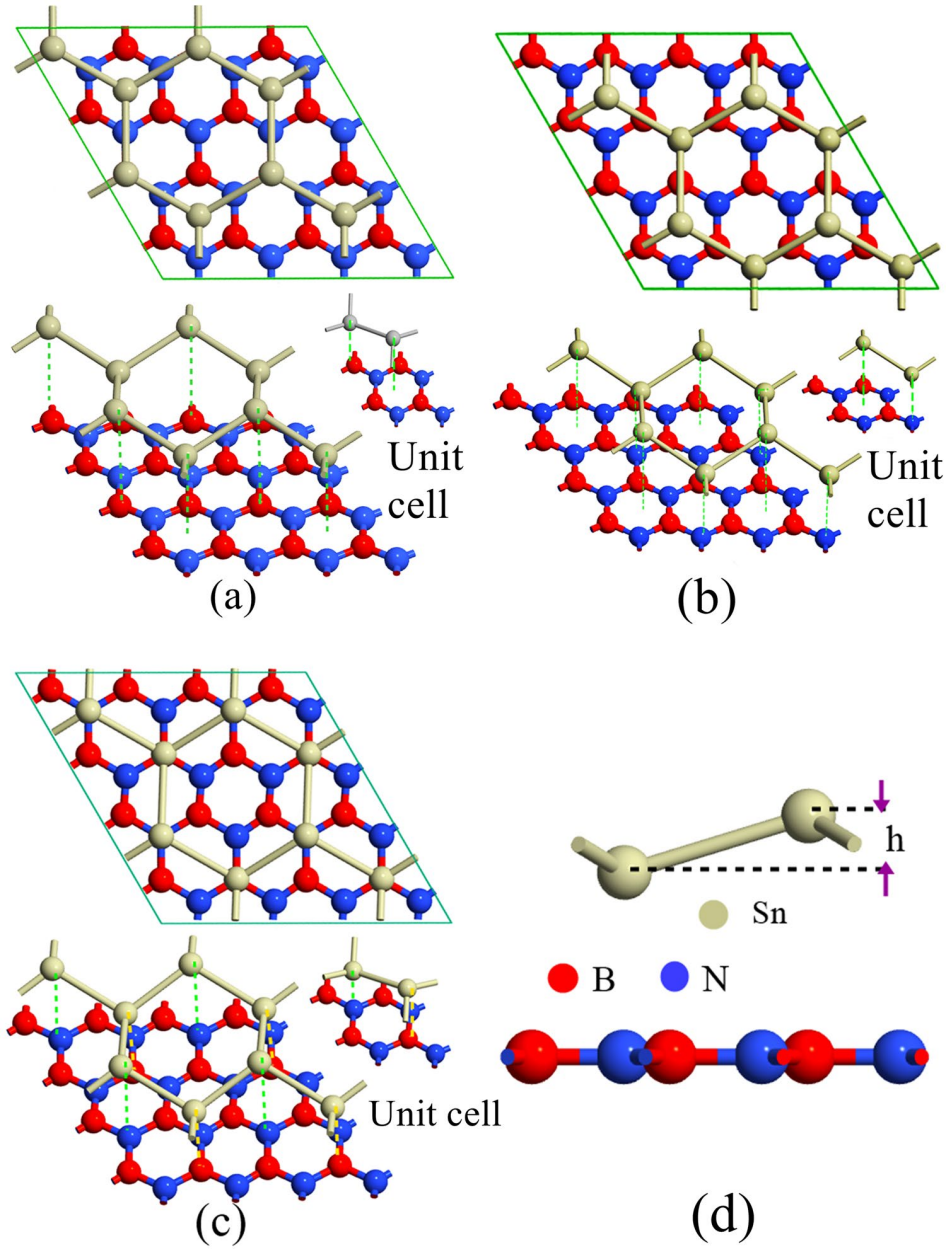
Atomistic model of Sn/h-BN heterobilayers with different stacking patterns. (**a**) supercell of Pattern I (**b**) supercell of Pattern II (**c**) supercell of Pattern III (**d**) Front view of the unit cell. It has two Sn atoms, four B atoms and four N atoms. ‘h’ denotes the buckling height of the stanene layer at equilibrium, which is 0.82 Å.

In our study, the geometry optimized lattice constant, Sn-Sn bond length and buckling height of the stanene are 4.687 Å, 2.834 Å and 0.82 Å respectively, which is in good agreement with the reported experimental^8,24^ and simulation^5,13,14,25–27^ studies. This is also in close agreement with the reported theoretical studies by Liu *et al*.^11^, Nissimagoudar *et al*.^28^ as well as by Peng *et al*.^29^. Again, in our study after the geometry optimization the lattice constant of h-BN is 2.504 Å, which is also in close agreement with the previously reported studies^30–33^ in the literature. The optimized lattice constant of stanene is ~47% greater than that of h-BN and therefore we have considered stanene/h-BN heterostructures composed of 4 × 4 lateral periodicity of h-BN and 2 × 2 lateral periodicity of stanene, as shown in Fig. 1 (Fig. 1(a),(b) and (c)). This supercell would experience a lattice mismatch of ~7% and therefore, stanene monolayer is stretched by~7% to ensure the commensurability of the heterostructure supercell.

The basic unit cell consists of two Sn atoms, four B atoms and four N atoms. Our Sn/h-BN heterobilayer configuration can be compared to the reported literature on the graphene/MoS_2_ heterobilayer structures^34,35^. A 4 × 4 supercell of MoS_2_ with a 5 × 5 supercell of graphene was considered due to the 23% difference in the lattice constants between graphene (2.42 Å) and MoS_2_ (3.12 Å) and the graphene layer was further stretched by ~3% to meet the commensurability criterion. Also, Chen *et al*.^14^ investigated the electronic and optical property of graphene/stanene heterobilayers where the lattice constants of graphene and stanene differ by about 46%. Hence, they^14^ proposed a supercell composed of 4 × 4 graphene and 2 × 2 stanene and further stretched the stanene layer by 4.7% to form commensurable structures.

Three stacking patterns of stanene-hexagonal boron nitride (Sn/h-BN) have been considered in this study as shown in Fig. 1(a),(b) and (c), respectively. Pattern I exhibits the heterobilayer structure with alternating Sn atoms either right over B atoms or on the top of the centers of BN hexagons. In case of pattern II, alternating Sn atoms are either over the N atoms or on the top of the h-BN centers. In case of pattern III, each of the Sn atoms lies either over the N atom or over the B atom. Similar stacking patterns are reported in the literature for the study of the heterobilayer structures such as graphene/h-BN^15,36^, graphene on copper substrate^36^, germanene/BeO^37^ and graphene/stanene^14^.

**Figure 2:**
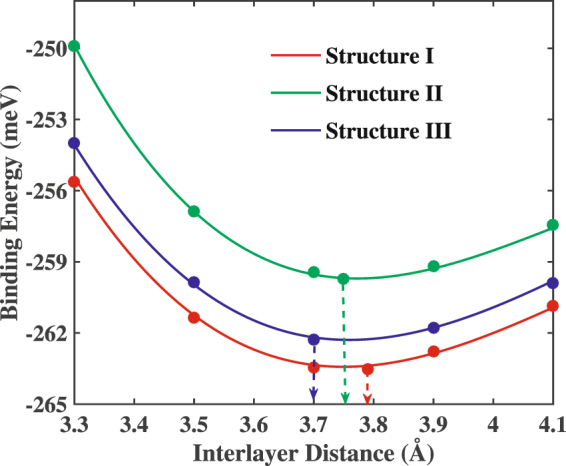
Binding energy per unit cell for three stacking patterns of Sn/h-BN heterobilayer with the variation of interlayer distance. The calculated equilibrium interlayer distances are shown by vertical arrows.

Figure 2 represents the binding energies per unit cell with the variation of the interlayer distance for the three considered configurations. Obtained equilibrium distances are 3.75 Å, 3.78 Å and 3.7 Å for structure I, II and III, respectively. For the benchmarking of our calculation, we have computed the optimized interlayer distance of the graphene/h-BN heterobilyer. Our computed equilibrium interlayer distance using DFT for the graphene/h-BN heterobilayer is 2.53 Å, which is in close proximity with the reported value (2.58 Å) by Ukpong *et al*.^32^ for graphene/h-BN heterobilayer. Our obtained equilibrium distances are also comparable to the reported^38^ interlayer distances (3.57 Å to 3.75 Å) for bilayer h-BN using GGA in the scheme of PBE. It is also comparable to the experimentally obtained interlayer distance of the Sn bilayers (3.5 Å ± 0.2 Å) during the epitaxial growth of stanene^8^ as well as with the calculated interlayer distance of the Sn bilayers (3.6 Å) by Evazzade *et al*.^39^ using GGA and PBE exchange correlation in the Quantum Espresso package where DFT-D modeling has been employed. On the other hand, vdW-DF2 has been reported to yield an equilibrium interlayer distance higher than the experimentally determined interlayer distance for some heterobilayers (3.77 Å for graphene-SiC^32^). In fact, the equilibrium interlayer distance is found to be sensitive with the choice of the exchange-correlation functionals^32,40^.

Moreover, as suggested by the binding energies of the three configurations shown in Fig. 2, all the considered configurations of the stanene/h-BN heterobilayers are electronically stable while structure I having the lowest binding energy. This can be attributed to the position of the B cations right under the Sn atoms of the stanene layer where π electron density is high, thereby enhancing the cation-π attractive interaction. On the contrary, in structure II, the N anions lie directly under the Sn atoms where the anion-π repulsive interaction occurs, thereby leading to a higher binding energy^15^.

Furthermore, the calculated binding energies in our study for the Sn/h-BN heterobilayers per unit cell at equilibrium are more than 250 meV which is higher^14,41^ than the binding energies of the weak vdW interactions^42^. This indicates that the stanene monolayer is bound to the h-BN substrate via interactions somewhat stronger than the weak vdW interactions^43^. The heterobilayers such as graphene/h-BN^15,36^, stanene/MoS_2_^44^ and graphene/SiO_2_^42^ are reported to have weak vdW interactions between the heterobilayers with a binding energy of less than 100 meV per unit cell. On the other hand, in the graphene/HfO_2_ heterobilayer structures, the graphene is strongly bound to the HfO_2_ with a binding energy of 110 meV^43^. The similar phenomenon has been reported in the study of the graphene/stanene heterobilayers^14^ with a binding energy of more than 180 meV per unit cell at optimum spacing. As an aside, the optimized interlayer distances for the three stacking configurations of the Sn/h-BN heterobilayers considered in this study are 3.7 Å to 3.78 Å, which are larger than the typical bond length of the Sn-B bonds (2.24 Å)^13^ and the bond length of the Sn-N bonds (2.14 Å)^13^. This indicates that, the Sn-B and the Sn-N covalent bonds are absent in the Sn/h-BN heterostructures and further conforms to the similar phenomena reported regarding the graphene/stanene^14^ and the graphene/HfO_2_^43^ heterobilayers.

**Figure 3:**
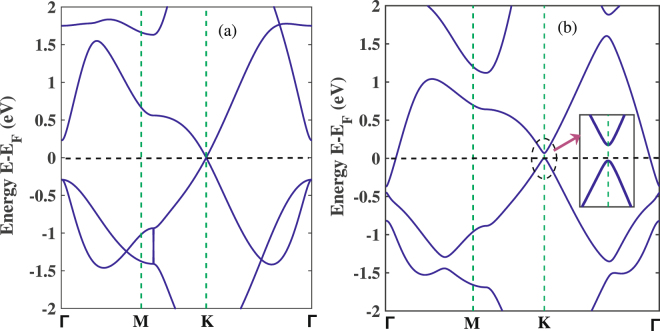
Band structures of stanene (**a**) without SOC and (**b**) in the presence of SOC.

For further benchmarking, we have computed the band structure of the 2D monolayer stanene without SOC as well as in the presence of SOC as shown in Fig. 3(a) and (b) respectively. As can be seen from Fig. 3(a), 2D stanene is a zero band gap material in the absence of SOC. When SOC is considered, a direct band gap of 69 meV (Fig. 3(b)) is opened at the K point. This is in excellent agreement with the DFT simulation study of 2D monolayer stanene using GGA in the scheme of PBE by Modarresi *et al*.^45^ (70 meV direct band gap in the presence of SOC) as well as by Xiong *et al*.^46^ (73 meV direct band gap with SOC), thereby confirming the validity of our simulations. Furthermore, in the presence of SOC a Fermi-level band crossing phenomenon of bands is observed at gamma (Γ) point. For the band structure of stanene as well as stanene nanoribbons in the presence of SOC, at gamma point similar phenomenon of either bands touching or crossing the Fermi level has been reported by Broek *et al*.^5^, Modarresi *et al*.^45^, Lu *et al*.^47^, as well as by Nagarajan *et al*.^48^. Despite this phenomenon, in view of the opening of the band gap at K point Broek *et al*.^5^ concluded that, pristine stanene offers perspectives of an all-Sn field effect transistor (FET). However, as discussed by Xiong *et al*.^46^ and Nagarajan *et al*.^48^, the effect of SOC on the band structure of stanene and the Fermi level touching or crossing phenomenon indicates its possibility of applications in spinelectronics and quantum spin Hall fields. Hence, the overall effect of SOC in the band structure of stanene and stanene based nanostructures requires consideration in terms of their promising application in nanoelectronics, spinelectronics, quantum spin Hall insulators as well as field effect transistor.

**Figure 4:**
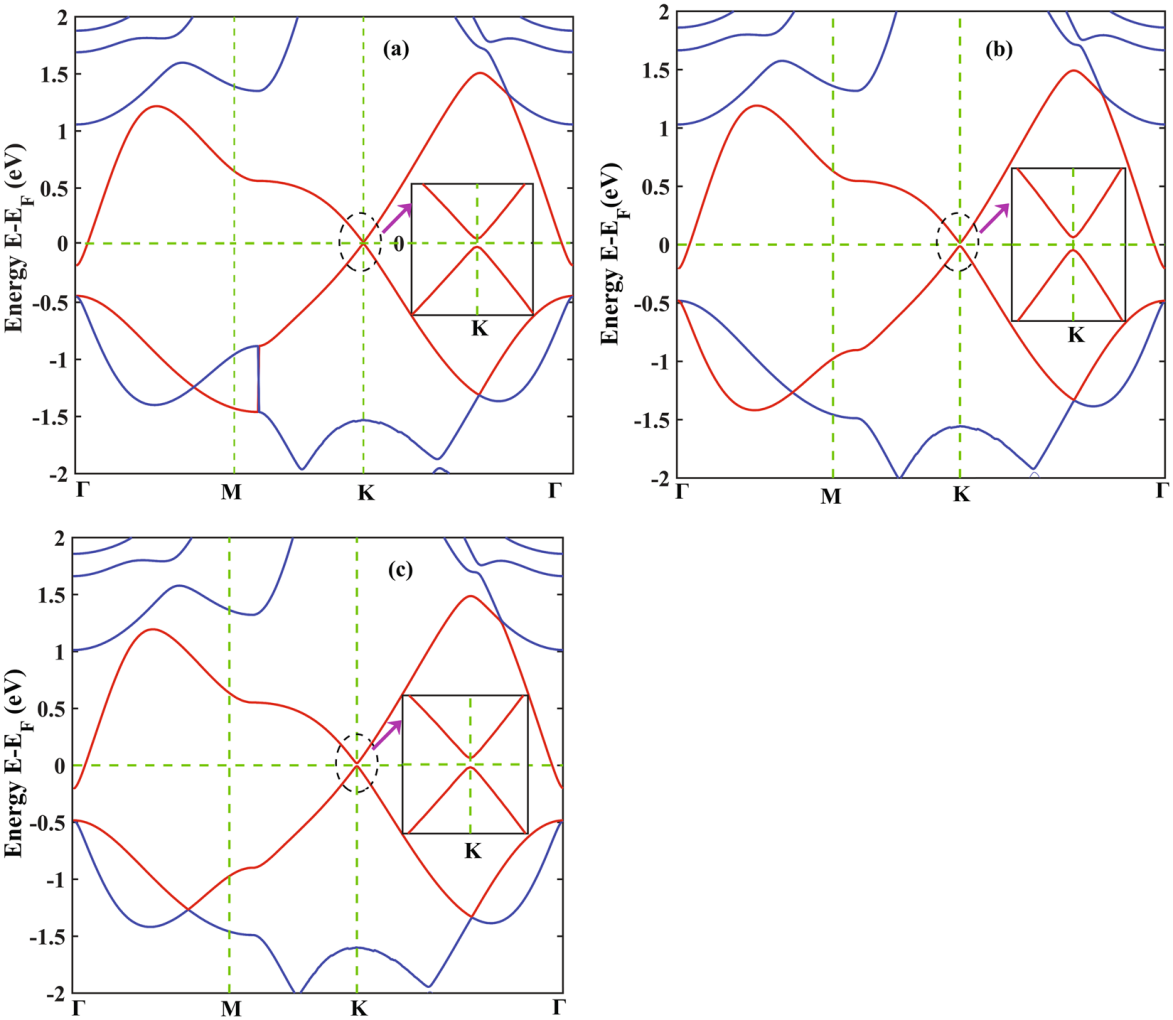
Band structures and the magnified band structures around K point of stanene/h-BN heterostructure without SOC (**a**) structure I (**b**) structure II (**c**) structure III.

Figure 4 shows the band structure of the stanene/h-BN heterobilayer without considering the effect of SOC for the three optimized configurations. The Fermi energy level is set to 0. The band structure patterns are almost similar for all the configurations and an opening of direct band gap of 25 meV for structure I, 33 meV for structure II and 30 meV for structure III can be observed at the K point. The magnified band structures around K point near the Fermi levels are also shown at the inset. As shown in the figure, linear Dirac dispersion relation is well preserved in all the three structures. Without SOC, pristine stanene shares the similar Dirac feature and no band gap is observed while the studied stanene/h-BN heterobilayer shows a direct band gap that indicates its possible application in nanoelectronic devices such as FETs.

**Figure 5:**
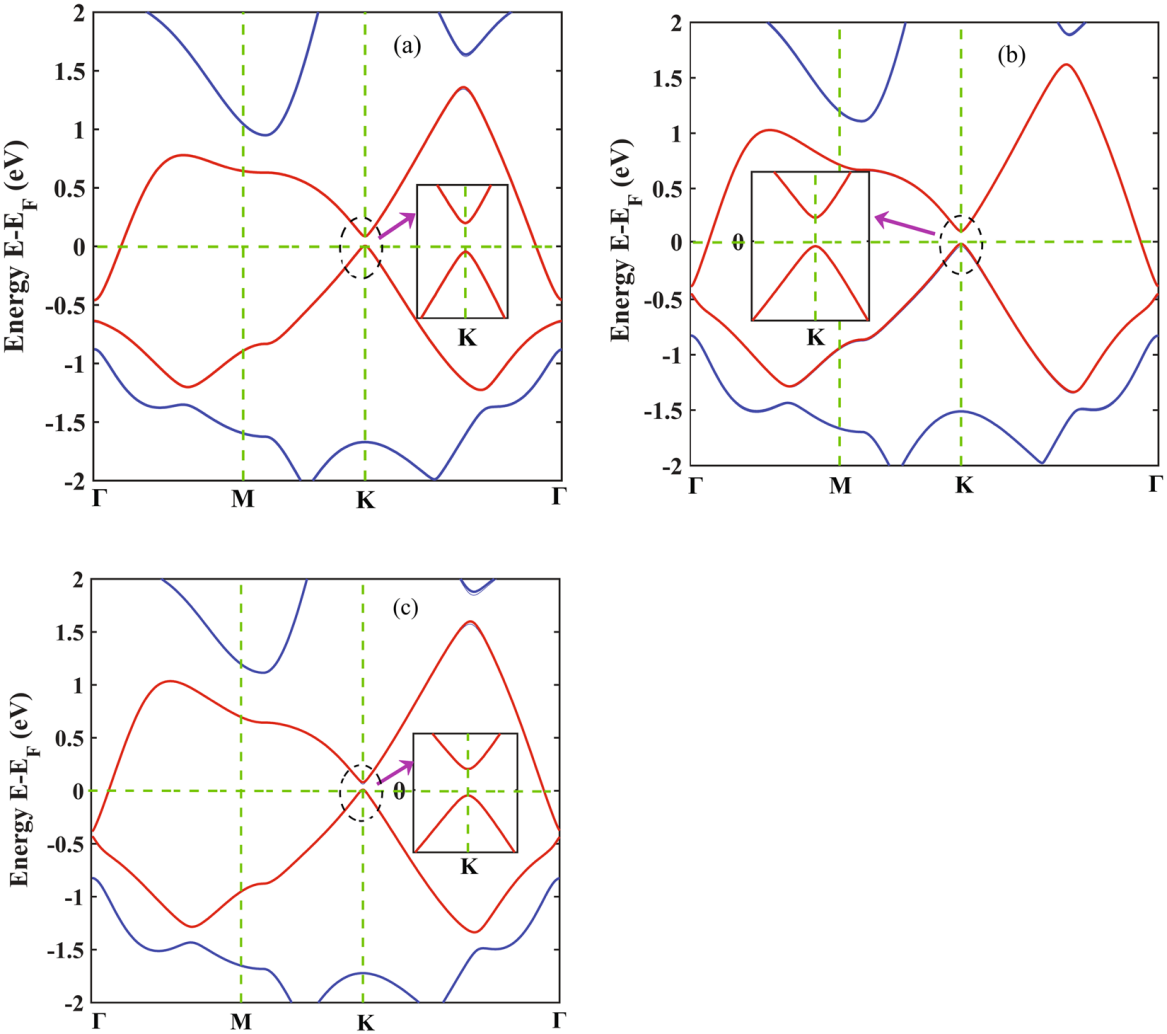
Band structures and the magnified band structures around K point of stanene/h-BN heterostructure with SOC for (**a**) structure I (**b**) structure II (**c**) structure III.

With the inclusion of SOC, the observed band gap for the structure I, II and III are 74 meV, 109 meV and 90 meV, respectively as shown in Fig. 5. The estimated band gaps including the effect of SOC are higher in magnitude with no significant change in the band structures near the K point as can be observed from the magnified plots in Fig. 5. This can be attributed to the effect of SOC on the heavy Sn atoms. In fact, group IV materials such as silicon (Si), tin (Sn) and lead (Pb) with single layer hexagonal configurations show SOC-induced band gaps and this effect gets stronger with the increasing atomic number of heavier atoms^49^. However, at gamma point (Γ) a Fermi-level band crossing phenomenon of bands can be observed which is similar to pristine stanene with the effect of SOC indicating the possibility of applying Sn/h-BN heterobilayers in the application of spinelectronics and quantum spin Hall insulators^46,48^.

The linear Dirac disperison near K point suggests a high charge carrier mobility. To further analyze this fact, we have calculated the effective mass of electrons $({m}_{e}^{\ast })$ and holes $({m}_{h}^{\ast })$ at the Dirac point for the three (3) configurations of stanene/h-BN heterobilayer. For structure III, the calculated effective mass for the electron ${(m}_{e}^{\ast })$ and the hole $({m}_{h}^{\ast })$ are 0.0507 ${m}_{0}$ and 0.0543 *m*_o_ respectively, which are small enough to provide high carrier mobility. The computed electron effective masses for the other two configurations are 0.054 ${m}_{0}$ (structure I) and 0.0513 ${m}_{0}$ (structure II). On the other hand, the calculated Fermi velocities are 0.538** × **10^6^ ms^−1^, 0.526** × **10^6^ ms^−1^ and 0.538** × **10^6^ ms^−1^ for structure I, II and III, respectively which are in the similar order of magnitude to that of stanene^5^ (0.97** × **10^6^ ms^−1^) and graphene^50^ (10^6^ ms^−1^).

Due to the interaction between the stanene and the h-BN layer, the sublattice symmetry of the zero band gap hexagonal stanene structure is broken, therefore opening a direct band gap. Similar phenomenon has been reported by Xiong *et al*.^51^ for the stanene/MoS_2_ heterostructure where the strong interaction breaks the sublattice symmetry of stanene and opens a band gap. Similarly, the strong interaction with the BeO monolayer substrate introduces a finite band gap in the germanene/BeO heterostructure by breaking the symmetry of the germanene^37^. Also, due to the symmetry breaking of the two carbon atoms in the hexagonal graphene induced by the interaction with the h-BN monolayer, a finite band gap of ~50 meV opens as reported in the literature^15,36^.

**Figure 6:**
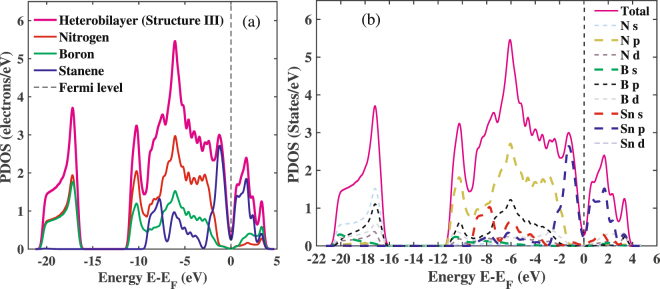
Density of states for structure III of the stanene/h-BN heterobilayer. (**a**) Total contribution from Sn, N and B atoms separately (**b**) Separate contributions from each orbital of the atoms.

**Figure 7:**
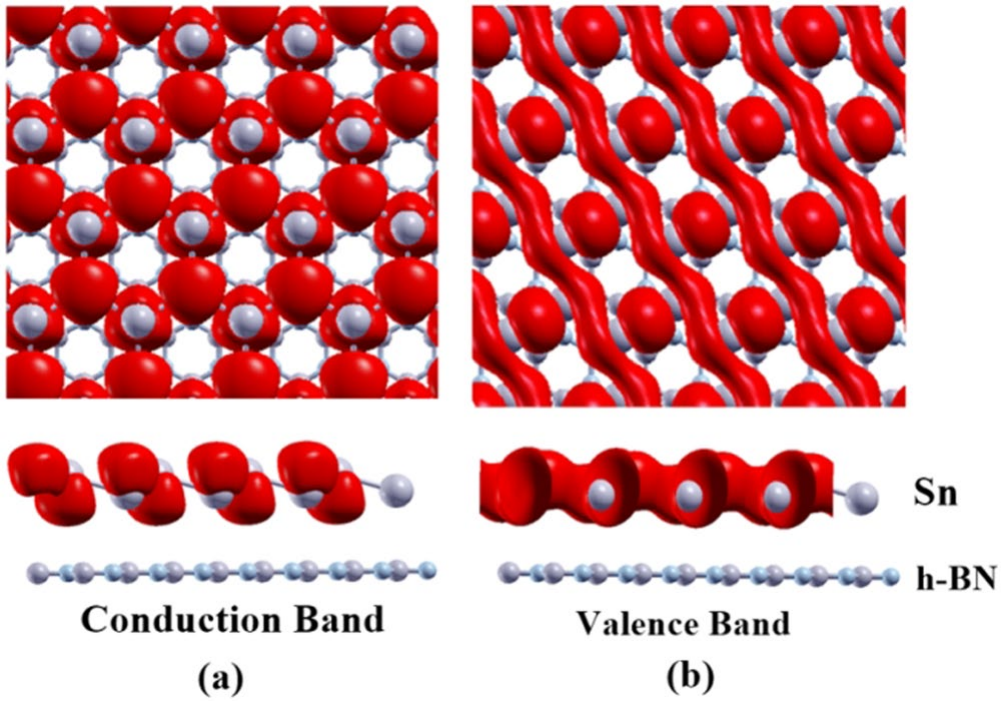
Real space charge density distribution of (**a**) conduction band and (**b**) valence band for structure III of the Sn/h-BN heterobilayer. The isovalue is 0.001 e/Å^3^.

Next, to analyze the electronic properties and gain deeper insight on the interlayer interactions, we have plotted the total as well as the projected density of states (PDOS) for structure III of the stanene/h-BN heterobilayer as shown in Fig. 6. As all the considered configurations of the Sn/h-BN heterobilayer are found to show similar band structure along with nearly same energy band gap, effective mass as well as Fermi velocity, hence we show the results for structure III only as a representative of Sn/h-BN heterobilayer. As depicted in Fig. 6(a), the peaks of the PDOS in the conduction band (0 to 2 eV) as well as in the valence band (−2 to 0 eV) are dominantly contributed by stanene. Again, molecular orbital resolved density of states presented in Fig. 6(b) reveals the dominant role of the π orbital of stanene in the conduction and valence bands, which is similar to the characteristics of the isolated monolayer stanene^5^. Furthermore, as shown in Fig. 6, unlike hybrid systems with high chemical interactions^52^, h-BN orbitals do not hybridize with stanene orbitals near the Fermi level ensuring non-intensive interactions of the two layers. This is further supported by the real space charge density distribution of the conduction and valence bands for structure III as shown in Fig. 7. The localization of stanene in the conduction as well as in the valence band of the heterobilayer structure further confirms the dominant role of stanene in shaping the electronic properties of the stanene/h-BN heterostructure. Therefore, electronic carriers are expected to transport only through the stanene layer, thereby leaving the h-BN layer to be a good choice as a substrate for the heterostructure. The localization of stanene in the conduction band and the valence band of the Sn/h-BN heterobilayer structure in this study can be compared to the heterostructures formed by other group-IV mono atomic 2D compounds such as graphene and silicene with h-BN working as a substrate. As reported by Balu *et al*.^18^, the band structure for the graphene/BN bilayer is dominated by the bands associated with the carbon atoms near the Fermi level, with a band gap of ~100 meV at the Dirac point. Similarly, silicene is reported^53^ to dominate the electronic transport in the silicene/h-BN heterobilayers and the Dirac cone of the silicene layer is preserved after contacting the BN layer, which is in accordance with our study for the stanene/h-BN heterobilayers. In fact, the heterostructures consisting of single layer silicene and graphene sandwiched between h-BN bilayers also show a band gap opening and high carrier mobility while the silicene and graphene layer remain nearly unaffected by h-BN^54,55^. In addition, the periodic heterostructures with a graphene sheet sandwiched between four h-BN layers also show similar phenomenon^56^.

In this section, we focus our study to tune up the band gap of the Sn/h-BN heterostructure for its possible use in the high performance nanoelectronic and spintronic devices.

**Figure 8:**
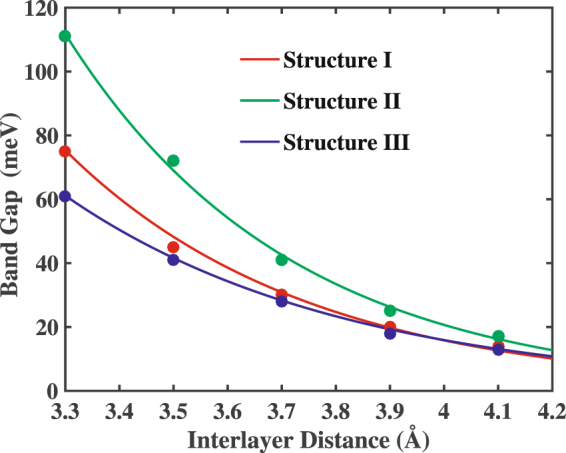
Variation in the energy band gap of Sn/h-BN heterobilayers as a function of interlayer spacing for the three different stacking configurations.

Firstly, we have calculated the energy band gaps with the variation of the interlayer spacing of the stanene and h-BN layers for all three considered configurations as presented in Fig. 8. With the increasing interlayer distance, energy band gap decreases. As the interlayer distance increases, the interaction between the h-BN and stanene layer decreases. As a result, the stanene layer tends to get back to its original symmetry^57,58^ therefore reducing the band gap. This is in line with the study of graphene/h-BN heterobilayers^15^ of various stacking patterns where the band gaps increase with the decrease in interlayer spacing.

**Figure 9:**
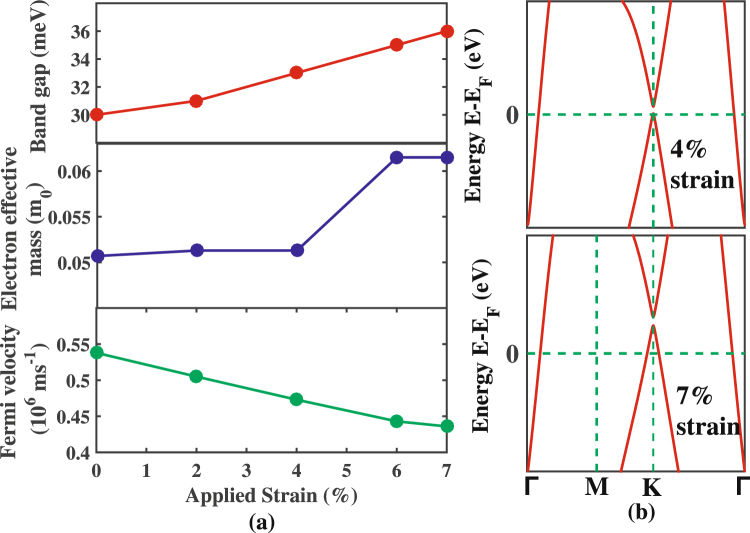
(**a**) Band gap, electron effective mass and Fermi velocity as a function of applied biaxial strain upto 7% for structure III of the Sn/h-BN heterobilayer. (**b**) Magnified band structures around K point near the Fermi level for 4% and 7% biaxial strain applied to the heterostructure.

Figure 9 shows the variation in the band gap for structure III under external strain of varying percentage. The strain $(\varepsilon )$ percentage is defined as the following:

2 $$\varepsilon =\frac{{a}_{strained}-{a}_{relaxed}}{{a}_{relaxed}}\times 100$$

where, a is the unit cell parameter. For the applied biaxial strain, the structural symmetry remains almost same as the unstrained case thereby causing a little increase in the band gap as shown in Fig. 9(a). The small changes in the effective mass and the Fermi velocities are also presented in Fig. 9(a). With the increasing percentage of strain, the Fermi level shifts downward and a self hole doping characteristics with hole carriers appear, which is evident in Fig. 9(b). In fact, at a strain of 4%, the Fermi level touches the valence band and further shifts downward at a maximum strain of 7%. This is also in line with the study for the stanene monolayer^5^. On the other hand, the reverse phenomenon i.e. the upward shift of Fermi level is expected for the compressive strain.

**Figure 10:**
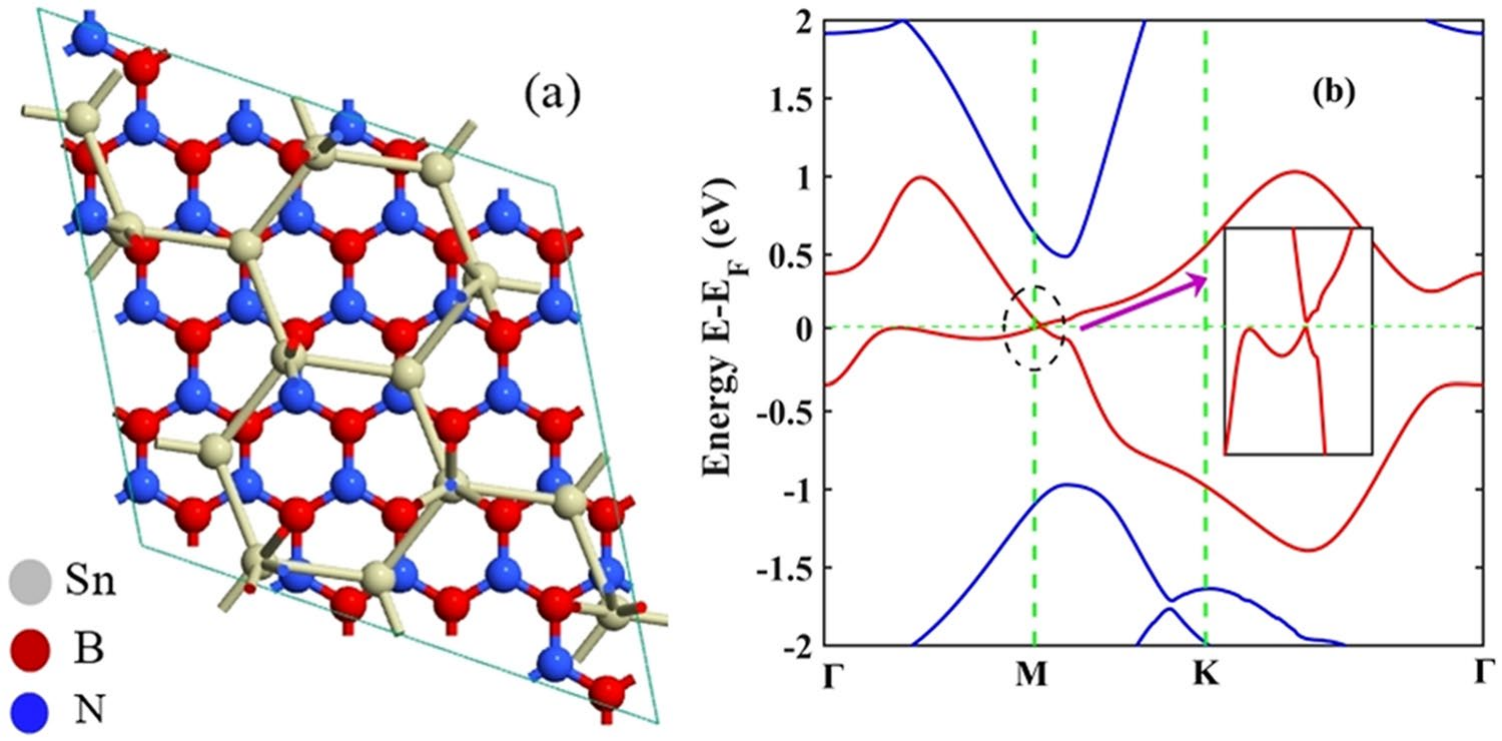
(**a**) Atomistic model of Sn/h-BN heterobilayer with a non-high symmetry configuration. (**b**) The electronic band structure and the magnified band structure without the effect of SOC around M point for the considered non-high symmetry configuration.

Finally, we have investigated one non-high symmetry configuration for the Sn/h-BN heterostructure as shown in Fig. 10(a). For the considered non-high symmetry supercell structure, the supercell is made up of √7 × √7 stanene and 5 × 5 h-BN and the stanene layer is rotated by $21.5^\circ$ with respect to the h-BN layer which ensures the commensurability condition in the supercell.

The electronic band structure for the non-high symmetry configuration is shown in Fig. 10 (b). A direct band gap of ~20 meV can be observed at M point which is comparable to the obtained band gap of ~30 meV for the high symmetry configurations of the Sn/h-BN heterobilayer in this study. Hence, it is reasonable to conclude that, non-high symmetry configurations will not substantially reduce the band gap of the Sn/h-BN heterobilayers. This is in accordance with the findings for graphene/h-BN heterobilayers as reported by Giovannetti *et al*.^36^.

## Conclusion

In summary, we have investigated the structural and electronic properties of the Sn/h-BN heterobilayers from DFT studies considering van der Waals interaction between two layers. We have considered three different stacking patterns and all of these are found to be electrically stable in terms of the high binding energy. The band structures of the Sn/h-BN heterobilayer shows a direct band gap of about 30 meV at the Fermi energy level while linear Dirac dispersion relation is closely maintained. The real space charge distribution and density of states of the heterostructure suggest the localization of stanene π orbital in the conduction as well as in the valence bands, further confirming the superior role of stanene in shaping the electronic properties of the stanene/h-BN heterostructure. Moreover, our calculated small effective mass and the emergence of Dirac cone suggest high charge carrier mobility for the Sn/h-BN heterostructure. Furthermore, our analysis concludes that, the band gap can be effectively tuned by varying the interlayer spacing between the Sn and h-BN monolayer while preserving the stability. A small increase in the band gap is observed with the increasing percentage of tensile biaxial strain. At a higher percentage of strain, the Fermi level shifts downward and self hole doping characteristics with hole carriers appear. Such unique and tunable electronic properties of the Sn/h-BN heterobilayer would further encourage the Sn-based nanoelectronic and spintronic device applications.
